# SEOM-GEINO clinical guidelines for grade 2 gliomas (2023)

**DOI:** 10.1007/s12094-024-03456-x

**Published:** 2024-04-25

**Authors:** María Ángeles Vaz-Salgado, Belén Cigarral García, Isaura Fernández Pérez, Beatriz Jiménez Munárriz, Paula Sampedro Domarco, Ainhoa Hernández González, María Vieito Villar, Raquel Luque Caro, María Luisa Villamayor Delgado, Juan Manuel Sepúlveda Sánchez

**Affiliations:** 1 Medical Oncology Department, Hospital Universitario Ramón y Cajal, Instituto Ramón y Cajal de Investigación Sanitaria (Irycis) CIBERONC, Madrid, Spain; 2 Medical Oncology Department, Complejo Asistencial Universitario de Salamanca, Salamanca, Spain; 3 Medical Oncology Department, Hospital Alvaro Cunqueiro-Complejo Hospitalario Universitario de Vigo, Pontevedra, Spain; 4 Medical Oncology Department, HM Universitario Sanchinarro-CIOCC, Madrid, Spain; 5 Medical Oncology Department, Complexo Hospitalario Universitario de Ourense (CHUO), Orense, Spain; 6 Medical Oncology Department, Hospital Germans Trias I Pujol(ICO)-Badalona, Instituto Catalán de Oncología, Barcelona, Spain; 7 Medical Oncology Department, Hospital Universitario Vall D’Hebron, Barcelona, Spain; 8 Medical Oncology Department, Hospital Universitario Virgen de las Nieves, Instituto de Investigación Biosanitaria Ibs.Granada, Granada, Spain; 9 Medical Oncology Department, Hospital Universitario Ramón y Cajal, Madrid, Spain; 10 Neuro-Oncology Unit, HM Universitario Sanchinarro-CIOCC, Madrid, Spain; 11 Medical Oncology Department, Hospital Universitario 12 de Octubre, Instituto de Investigación 12 de Octubre (I+12), Madrid, Spain

**Keywords:** Low-grade glioma, Guideline, Neuro-oncology, Oligodendroglioma, Astrocytoma, IDH mutation

## Abstract

The 2021 World Health Organization (WHO) classification has updated the definition of grade 2 gliomas and the presence of isocitrate dehydrogenase (IDH) mutation has been deemed the cornerstone of diagnosis. Though slow-growing and having a low proliferative index, grade 2 gliomas are incurable by surgery and complementary treatments are vital to improving prognosis. This guideline provides recommendations on the multidisciplinary treatment of grade 2 astrocytomas and oligodendrogliomas based on the best evidence available.

## Introduction

Grade 2 gliomas, also called low-grade gliomas (LGG) are characterized by a low proliferation index, diverse pathology, and clinical behavior. Different treatment options include surgery, radiotherapy (RT), and chemotherapy (CT) and management calls for a multidisciplinary approach [1]. IDH inhibitors are new treatments that have recently shown significant efficacy in grade 2 gliomas not previously treated with radiotherapy or chemotherapy [2]. Most clinical trials have addressed the timing of RT and CT; however, these historical studies enrolled patients with different pathologies according to the new classification and molecular markers such as IDH and 1p19q [3], that are essential for the diagnosis, were not taken into account when they were designed. Clinical guidelines are important to bring order to the evidence available and help multidisciplinary teams plan treatments.

## Incidence and epidemiology

Low-grade gliomas (LGG), as per World Health Organization (WHO) include grade 2 oligodendrogliomas and astrocytomas [3]. A recent registry shows that LGG account for 5–10% of all primary brain tumors. The incidence rate of primary brain and nervous system tumors in adults in the United States is approximately 30 per 100,000 persons, and approximately 2–3 per 100,000 are LGG [4]. These guidelines will focus on grade 2 gliomas, for which IDH mutations are a milestone genetic alteration.

Most adult primary brain tumors are sporadic with no identifiable risk factors. Nevertheless, a small proportion of brain tumors have been linked to rare genetic syndromes, such as neurofibromatosis, von Hippel Lindau, and Li-Fraumeni [5].

Other risk factors analyzed include cell phones and radiofrequency fields, including microwave, radar equipment, and occupational exposures, which are difficult to quantify. The results of numerous epidemiologic studies have failed to be conclusive. A meta-analysis that included data from 22 case–control series settled that there was a slight increase in risk associated with cell phone use, but there were potential confounding factors, and only case–control studies were involved [6]. Another conclusion of the meta-analysis is the fact that risk appeared after an induction period of 10 years or more.

On the other hand, the association between forms of nonionizing radiation and cancer is less clear, and the data do not support an important role [7, 8].

An established risk factor for primary brain tumors is exposure to ionizing radiation. Cohort studies of atomic bomb survivors and childhood cancer survivors have demonstrated that cranial radiation is associated with an increased risk for a variety of brain tumors, including meningiomas, gliomas, and nerve sheath tumors [9].

## Methodology

**Fig. 1:**
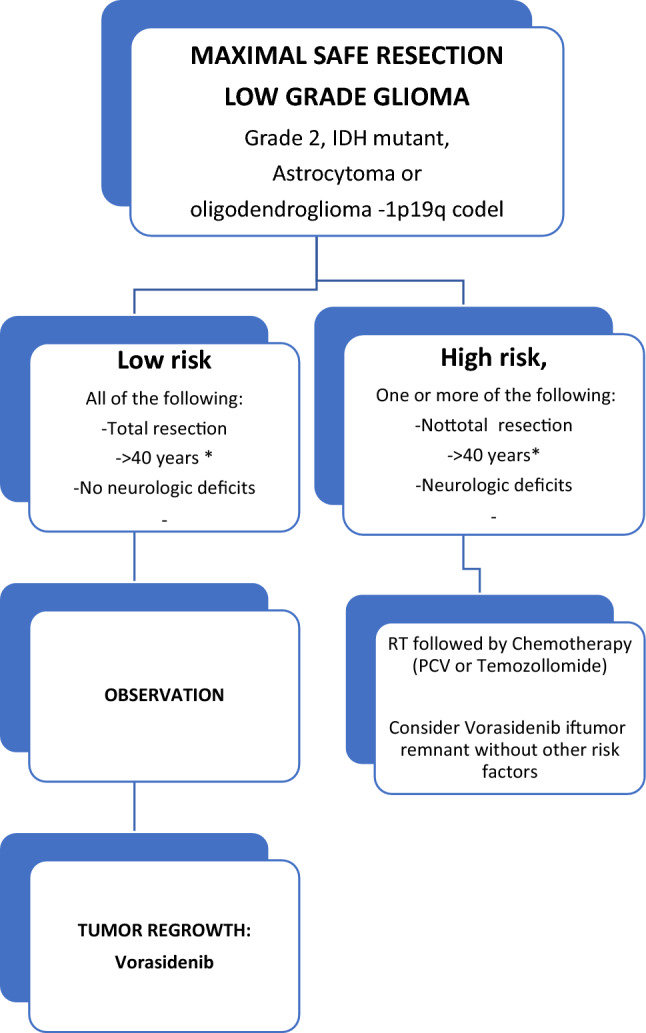
Recommendations of LGG management after surgery. Asterisk: age < 40 is strongly associated with the presence of IDH mutations and may not be an independent prognostic factor, but a surrogate marker of IDH

The aim of this document is to provide a clear, practical recommendation for the management of grade 2 gliomas in Spain. These guidelines have been elaborated by a multidisciplinary group with expertise in clinical and investigational neuro-oncology. A bibliographic search of the MEDLINE database (PubMed) was conducted. The different sections were drafted by different responsible experts; all the authors subsequently discussed the results and determined the level of evidence reported in Table 1 [10] (Fig. 1).

**Table 1:** Levels of evidence/ grades of recommendation

Levels of evidence
I. Evidence from at least one large randomized, controlled trial of good methodological quality (low potential for bias) or meta-analyses of well-conducted randomized trials without heterogeneity
II. Small randomized trials or large randomized trials with a suspicion of bias (lower methodological quality) or meta-analyses of such trials or of trials with demonstrated heterogeneity
III. Prospective cohort studies
IV. Retrospective cohort studies or case–control studies
V. Studies without a control group, case reports, expert opinions
Grades of recommendation
A. Strong evidence for efficacy with a substantial clinical benefit; strongly recommended
B. Strong or moderate evidence for efficacy, but with a limited clinical benefit; generally recommended
C. Insufficient evidence for efficacy or benefit that does not outweigh the risk or the disadvantages; optional
D. Moderate evidence against efficacy or for adverse outcome; generally not recommended
E. Strong evidence against efficacy or for adverse outcome; never recommended

## Diagnosis, pathology, and molecular biology

The cardinal symptom of LGG is the presence of adult-onset seizures in 80% of the cases. Due to their slow growth rate and infiltrating nature, the presence of focal neurological symptoms is less frequent at diagnosis than in high-grade tumors, and in a significant percentage of cases, LGG can be diagnosed incidentally [11].

The 2021 WHO classification has clarified that the diagnosis of LGG should combine imaging, histopathology, and molecular diagnostic methods [3].

The radiological modality of choice for LGG is magnetic resonance. Typically, LGG are homogeneous and hypointense on T1 and hyperintense on T2 FLAIR sequences. Calcifications and the presence of contrast enhancement are more common in oligodendrogliomas, whereas T2–T1 mismatch suggests the presence of an astrocytoma. Certain findings, such as the presence of heterogeneity, increased perfusion, restricted diffusion, and widespread contrast enhancement, suggest the presence of a higher-grade component and a guided biopsy should be considered when complete resection is not feasible [12] (Level IV-B).

LGG are separated into astrocytomas and oligodendrogliomas. Those with uniformly rounded nuclei and perinuclear halo (“fried egg”) are considered oligodendrogliomas, while those with nuclear irregularities with fibrillary processes are diagnosed as astrocytomas) [12]. However, tumors having the same morphological characteristics do not always share molecular or clinical characteristics. In contrast with G3 gliomas (formerly called anaplastic gliomas), G2 gliomas must not present anaplasia or mitotic activity [13] (Level IV-B).

LGG should be evaluated using the most recent WHO Classification of Tumors of the Central Nervous System published in 2021 [14] (Level IV-A). All tumors with diffuse glioma morphology should undergo IDH and ATRX immunochemistry (IHC) staining. IDH1 and IDH2 sequencing is necessary for patients with negative IHC and grade 2–3 features and for grade 4 patients < 55 years (Level IV-A).

In tumors that are IDH mutant and ATRX wild type (WT), 1p-19q co-deletion is necessary to establish a diagnosis of oligodendroglioma, while IDH mutant –ATRX mutant patients can be diagnosed with IDH mutant astrocytoma.

In the current edition of the WHO classification, the presence of specific molecular alterations can overwrite the histopathological parameters and be sufficient to assign a higher grade [3]. For example, in an IDH-wildtype diffuse astrocytic tumor, the presence of TERT promoter mutation, EGFR gene amplification, or + 7/ − 10 chromosome copy number changes, is sufficient to diagnose IDH-wt Glioblastoma, grade 4, and the presence of CDKN2A/ B homozygous deletion results in a WHO grade 4 IDH-mutant Astrocytoma (Level IV-A).

Pediatric-type diffuse gliomas, in particular, diffuse LGG, MAPK pathway-altered, that can present astrocytic or oligodendroglial morphology should receive special attention, particularly in young adult patients, and the presence of mutations in genes such as BRAF, CRAF, NF1, FGFR1, NTRK2, or PTPN11, among others, can be identified and enable targeted therapies [15].

The 2021 WHO classification suggests that results should be provided using a layered report structure in which an overall “integrated” diagnosis is given and includes the histopathological denomination, grade, and molecular information. For instance, diffuse gliomas that occur in adults (adult-type gliomas) are given the integrated diagnosis of either astrocytoma, IDH-mutant (that can be grades 2,3 or 4); oligodendroglioma, IDH-mutant (that only can be grade 2 or 3), or IDH wt glioblastoma (G4) and then, the histopathological diagnosis, grade, and other molecular information is provided in the report [3].

## Staging and risk assessment

### Risk assessment

It is important to identify patients who may benefit from postoperative therapy, considering specific clinicopathological factors related to the high risk of recurrence. (Level I-A)*.* Classically, the Pignatti risk score (age ≥ 40 years, largest diameter of tumor ≥ 6 cm, tumor crossing the midline, astrocytic histology, and presence of neurologic deficit prior to surgery) has been taken into account. The presence of three or more of these risk factors identifies a high-risk patient group and is associated with poor outcomes and shorter survival [16].

The Radiation Therapy Oncology Group (RTOG) 9802 phase III trial included patients with a diagnosis of grade 2 glioma. This trial showed a survival benefit with the addition of CT to adjuvant RT in those individuals with newly diagnosed, high-risk, supratentorial WHO grade II gliomas. The two risk factors for disease progression that were used to assign postsurgical treatment were subtotal resection (STR) or age ≥ 40 years [1].

In the same trial, a phase II observational study was conducted in patients who were initially considered to be at low risk (< 40 years old and underwent complete surgery) and were not treated with radiotherapy or chemotherapy. In this group of patients who underwent observation without active treatment, three factors were identified that correlated with a worse prognosis: preoperative tumor diameter ≥ 4 cm, astrocytoma/oligoastrocytoma histological subtype, and residual tumor ≥ 1 cm according to MR imaging [17]. However, it is important to note that age < 40 is strongly associated with the presence of IDH mutations and may not be an independent prognostic factor, but a surrogate marker of IDH.

The previously mentioned prognostic factors are based on morphological criteria; nevertheless, at present, the molecular study must also be factored in, given the 2021 WHO Classification of Tumors of the Central Nervous System [3]. The presence of an IDH mutation is the most important molecular marker and is associated with favorable prognosis and increased overall survival (OS) [18, 19]. Likewise, 1p19q co-deletion correlates with both greatly improved progression-free survival (PFS) and OS [20, 21].

In conclusion, the post-surgical low-risk patient group are those in which we can find all of the following characteristics: ≤ 40 years, gross total resection (GTR), and IDH-mut, 1p19q-co-deletion tumor (Level II-B).

The *high-risk* patient group, who would benefit from adjuvant treatment, are > 40 years, have STR, or biopsy without IDH mutation (Level I-A).

Other risk factors to take into account are neurologic deficits and preoperative tumor diameter (Table 2).

**Table 2:** High-risk factors to consider adjuvant therapy in low-grade gliomas

High risk factors	
Clinical	Major risk factors: Age > 40 years Not total resection -Partial resection, STR or biopsy- Others: Neurologic deficits Tumor size ≥ 6 cm Tumor crossing the midline
Molecular	IDH wild type 1p19q non-co-deleted

## Grade 2 glioma management surgery

### Maximal resection surgery versus biopsy

Surgical resection is the first step in the diagnosis of LGG, even in incidentally discovered tumors. The goal of surgery should be to remove as much tumor as possible [22, 23] (Level IV-B) whenever feasible and safe and does not compromise neurological function. While there are no randomized trials that have established the benefit of maximal surgical resection compared to more limited resection, we have data from retrospective studies and secondary analyses of large trials and meta-analyses that demonstrate that the type of resection is an independent prognostic factor for PFS (Level III) and OS (Level III) [24–28], as well as helping to control symptoms such as seizures, reducing mass effect, and the risk of intracranial hypertension [22, 29–32] (Level III). On the other hand, an adequate sample enables the diagnosis and correct molecular analysis to be made.

Despite these data, in some cases, a biopsy is the only thing that needs to be considered [33] (Level IV-B):

- In the case of preoperative estimated resection of less than 50%.
- When the diagnosis is necessary for deep lesions (including the brain stem).
- In the case of a diffuse and/ or multicentric tumor
- In the event of any contraindication to open resection.

The biopsy can be stereotactic or open, although frameless neuro-navigated biopsy is an option to be considered [34, 35] (Level IV-B).

### Incidental LGG

The prevalence of silent LGG is estimated to be between 0.02 and 0.09% and the reported rate of incidentally diagnosed gliomas ranges between 3% and 10.4%, depending on the series consulted [36–41].

Therefore, regardless of whether it is incidental or not, efforts to obtain complete resections are justified as long as doing so does not compromise the patient’s quality of life. Certain minor expected deficits (such as quadrantanopia) might be deemed acceptable, but only after a shared decision-making process with the patient (Level IV-B) [42].

### Technical tools to support surgery

Preoperative MRI with diffuse tensor imaging improves postoperative outcomes (Level III-B) in lesions near the motor and sensory tracts. Neurophysiologic evaluation, intraoperative mapping, and awake surgery are advantageous in tumors located in eloquent areas to decrease postoperative morbidity [43] (Level III-B).

### Contribution of molecular classification to surgery

Regarding the WHO molecular classification, while extensive resection benefits all molecular subtypes, it appears to be more relevant in IDH-mutated astrocytomas, in which even limited residual disease can have a negative impact on OS compared to complete resection [24, 43] (Level III).

## Radiotherapy (RT)

RT is deemed part of standard management for LGG. RT trials have shown that it can increase PFS and reduce symptoms (especially epilepsy) without an increase in OS [44].

There is controversy about the best time to administer radiotherapy treatment, the dose, and the schedule, depending on the grade of the tumor.

The EORTC 22845 trial compared early versus delayed RT in LGG. Median PFS was 5.3 years in the group receiving early RT and 3.4 years in the group in whom it was delayed; nevertheless, OS was similar in both groups, 7.4 years [45]. Early RT was administered in the immediate postoperative period between three and five weeks after surgery.

The administration of increased radiation doses offers no clear advantage and lower doses of RT (45–54 Gy) are recommended for LGG, including high-risk cases [45]. However, for IDH wild-type low-grade gliomas, which have similar survival rates to high-grade gliomas without the IDH mutation, a dose of 59.4 to 60 Gy can be considered for this subgroup of patients with the worst prognosis.

Alternative RT approaches have included proton RT, hypofractionated RT, and fractionated stereotactic RT. These are still of recent use in the treatment of gliomas and studies have shown no differences with respect to conventional radiotherapy. Preliminary data from a study in pediatric patients reveal that proton therapy might reduce radiation dose to developing brain tissue and potentially decrease toxicities without compromising disease control [46].

Although RT is well tolerated, it has both short- and long-term side effects. Perhaps most salient in the short term are hair loss, asthenia, loss of appetite, and headache.

We can conclude that immediate radiotherapy, without chemotherapy, lengthens the period without progression, but does not impact OS. High-risk patients or those with symptomatic gliomas (seizures) should be the ones to be considered for immediate radiation therapy. The administered dose should be between 45 and 54 Gy, except for very high-risk tumors, such as IDH wild type, which may require doses of up to 60 Gy (Level I-B).

## Systemic treatment

The standard of care for patients who are eligible for postsurgical treatment has been RT followed by PCV chemotherapy (procarbazine, lomustine (CCNU), and vincristine) (Level I-A). This regimen is based on the phase III RTOG 9802 trial [1] in which 251 patients were randomized, from 1998 to 2002, into RT alone versus RT and PCV (six cycles). The endpoint was OS. Suitable patients had high-risk factors: incomplete resection and/ or age ≥ 40 years. The trial population included individuals with astrocytoma, oligoastrocytoma, and oligodendroglioma. No molecular analysis was carried out. RT consisted of 54 Gy in 30 fractions of 1.8 Gy each. Chemotherapy consisted of six cycles of PCV every 8 weeks (Table 3). Median follow-up was 11.9 years; 55% of the study sample died. The addition of PCV demonstrated a clear benefit with nearly double OS (13.3 vs. 7.8 years, HR 0.59; *p* = 0.003) and prolonged PFS (10.4 vs 4 years, HR 0.50, *p* < 0.001). The observed benefit was most definitive for oligodendrogliomas. Adverse effects were greater in the group that received CT. The addition of PCV was not detrimental to cognitive function relative to RT alone. In a limited analysis, post hoc treatment with post-radiation CT was associated with longer OS and PFS in the IDH-mutant subgroups, and no significant difference was observed in the IDH-wild-type subgroup [47].

**Table 3:** Chemotherapy schemes

PCV
CCNU-lomustine: 110 mg/ m^2^ day 1, p.o
Procarbazine: 60 mg/ m^2^, days 8–21, p.o
Vincristine 1.4 mg/ m^2^, days 8 and 29, iv (max dose 2 mg) every 8 weeks
Dose-dense temozolomide
75 mg/ m^2^ days 1–21, every 28 days, p.o

There are no studies comparing CT versus the combination of RT and CT. Nevertheless, a phase 3 intergroup study (EORTC 22033–26033) did compare RT and CT. Patients aged 18 years or older who had a low-grade glioma with at least one high-risk characteristic (aged > 40 years, progressive disease, tumor size > 5 cm, tumor crossing the midline, or neurological symptoms) were enrolled [48]. A total of 477 patients were randomized to receive RT (up to 50·4 Gy) or dose-dense oral temozolomide. The endpoint was PFS. No significant differences were detected (46 months with radiotherapy and 39 months with temozolomide (unadjusted HR 1.16 [95% CI 0.9–1.5], p = 0.22)). More follow-up is required. CT alone can be contemplated if RT is not possible.

Vorasidenib is the first IDH inhibitor of mutant IDH1 and IDH2 enzymes that has showed significant improvement in PFS in patients with grade 2 IDH-mutant gliomas that had not received other previous treatment than surgery [2]. The INDIGO trial was a double-blind, phase 3 trial, that assigned 331 patients with residual or recurrent grade 2 IDH-mutant glioma to receive either vorasidenib or placebo. The primary endpoint was imaging-based PFS according to a blinded assessment by an independent review committee. The most important secondary endpoint was the time to the next anticancer intervention. Vorasidenib improved PFS (median: 27.7 months vs 11.1 months, HR: 0.26, p < 0.001) and the time to the next intervention (HR: 0.26; p < 0.001) without significant toxicity in this specific population.

### Recommendations

Following surgery, the standard of care for patients with high-risk grade 2 gliomas is RT followed by PCV polychemotherapy (Level I-A).

Vorasidenib is the treatment of choice in patients who were not treated with chemotherapy or radiotherapy, have a tumor remnant after surgery, or have had disease regrowth after surgery (Level I-A). Despite this evidence, Vorasidenib is still awaiting approval from FDA and EMA.

## Management of recurrent disease

There is no standard treatment for recurrent low-grade glioma except for those treated only with surgery in which vorasidenib has shown a significant improvement in PFS (Level I-A) [47]. Therapy at progression depends on Karnofsky performance status, neurological status, patterns of progression, and first-line therapy.

Second surgery should always be considered in recurrent low-grade glioma patients whose lesions are resectable (Level IV-C). Several studies have demonstrated that gross resection of lesions can be beneficial, due to decreased tumor load [27, 49] and improved overall survival [50]. Suitable comprehensive assessment is crucial for proper selection of candidates for the second surgery; an evaluation by a multidisciplinary committee is recommended [14, 51, 52]. Adjuvant treatment should be contemplated in subjects who have not previously received it (Level II-C).

For individuals who are not candidates for surgical rescue and have not received prior treatment, irradiation or CT are possible options [14] (Level III-C). Alkylating agent-based CT is often the mainstay of treatment for recurrent gliomas. Multiple previous trials have explored the role of TMZ in recurrent LGG and have reported response rates of some 50% and 6-month progression-free survival of 76–98% [53, 54]. Temozolomide is sometimes preferred over PCV because of its favorable safety profile and ease of administration [14] (Level III-C).

For patients resistant to both temozolomide and nitrosurea-based treatments such as lomustine of PCV, there is limited evidence of benefit with either bevacizumab or conventional chemotherapy regimens, and participation in clinical trials should be encouraged wherever possible (Level III–V).

Molecular profiling using NGS can help identify potential clinical trial options and should be offered especially as encouraging preliminary activity has been reported in clinical trials with IDH inhibitors in the refractory setting[55, 56], FGFR inhibitors [57, 58], panRAF inhibitors [59] and NTRK inhibitors [60] in patients with grade 2 gliomas (Level II-B).

Indications for re-irradiation remain controversial. In selected cases, if the new lesion is outside the target of the previous RT or the recurrence is small, it can be pondered (Level V-C) [61]

## Follow-up, long-term implications, and survival

Low-grade gliomas (LGG) often present long-term life expectancy thanks to multimodal therapeutic management. Prolonged surveillance is prudent to detect tumor growth and malignant transformation early before symptoms develop and neurological function is compromised. It allows for smaller radiation fields, safer surgical resection, less neurological morbidity, and, presumably, improved survival [62–64] (Level III-C).

There are no formal clinical trials that have defined the optimal frequency for follow-up after treatment. If possible, every brain tumor follow-up protocol should include the same technique to reduce variability in imaging interpretation [65] (Level III-C).

The National Comprehensive Cancer Network (NCCN) establishes that an MRI should be obtained for grade 2 gliomas every 3–6 months for 5 years, and then at least every 6 months or as clinically indicated (Level II-A) [61]. Nevertheless, a recent proposal has been put forth for surveillance imaging in newly diagnosed LGG and their recurrence that includes histological grade and molecular subtype (2021 WHO classification) considering growth rate and outcomes following the addition of CT and RT to surgery [66].

For WHO Grade 2 astrocytoma and oligodendroglioma, clinical trials have shown median time to tumor progression (mPFS) > 2 years after treatment initiation either with RT alone (4 years PFS in RTOG 9802 and 3.8 years in EORTC 22033), RT with CT (10.4 years in RTOG 9802), and CT alone (3.2 years in EORTC 22033) [1, 48]. Therefore, these data speak to not reducing close follow-up after 2 years, particularly in subjects who have not received RT or CT. In contrast, in a small number of patients with IDHmut who have received both RT and PCV, improved PFS was demonstrated > 50% after 10 years of treatment [1], suggesting that less intensive initial monitoring is reasonable.

Taken together, a rational approach is MRI monitoring every 6 months in cases on non-deletion and every 6–9 months for those with codeletion, especially if complete resection has been achieved, until the first tumor progression. In those patients treated only with surgery or those who received RT or CT alone, an MRI should be performed as often as every 3–4 months until tumor progression (Level II-B).

Despite prolonged survival, some LGG relapse and retrospective analyses reflect a shorter interval between the first (PFS1) and second progression (PFS2) compared to the initial diagnosis (3 years vs. 5.7 years, respectively) [67]. Therefore, the recommended follow-up is every 3–4 months after the first relapse (Level II-B).

In subjects with equivocal or suspicious MRI findings in the first 3 months after chemoradiation, a short interval between follow-up checks of 4 weeks is appropriate to determine progressive disease versus pseudo-progression. However, in patients with IDHmut, repeating MRI in 12–16 weeks is also reasonable, inasmuch as longer intervals may be required to understand the nature of changes (Level III-B).

For individuals who experience clinical changes (seizures, higher corticosteroid dosage, or clinical suspicion of tumor progression), an unscheduled MRI should be performed (Level III-B).

### Long-term implications and survival

Regarding health-related quality-of-life (HRQoL), LGG are most commonly diagnosed in working-aged adults and, given their long survival, QoL is of great interest in follow-up. Evidence suggests that these patients’ overall HRQoL is poor and stable for years. However, some studies have demonstrated significant improvement in emotional well-being at one or three years compared to 1 month since treatment [68].

In a systematic search of studies addressing HRQoL, the factors most often associated with worse QoL were cognitive dysfunction and fatigue, as well as epilepsy/ seizure burden [67]. Other functional impairments, such as communication deficits, pain, and headaches are frequently observed. Similarly, emotional, physical role, and social functioning impact general and mental health perception, in addition to vitality, compared to non-cancer controls. Due to treatments and tumor location, patients with LGG can experience problems with self-care; thus, different support self-management programs tailored to this patient population comprise a good strategy to improve QoL outcomes [69].

With respect to seizure development in LGG patients who have undergone resection, a recent, retrospective review has revealed that complete and near-total resection (> 90%) correlate with improvement in seizure control compared to subtotal resection (p = 0.066). Future prospective studies investigating the efficacy of prophylactic and maintenance antiepileptic therapy in subgroups of glioma patients are needed before generalizing their use to routine clinical practice [70] (V-C). The summary of recommendations is shown in Table 4.

**Table 4:** Summary of recommendations

Risk assessment
The post-surgical low-risk LGG patient are those in whom we can observe all of the following characteristics: ≤ 40 years, gross total resection (GTR), and IDH-mut, 1p19q-codel tumor (Level II-B)
The high-risk patient group, i.e., those who could benefit from adjuvant treatment, are > 40 years, had STR, or biopsy without IDH mutation (Level I-A)
Surgery
Surgical resection is the first step to diagnose LGG, even in incidentally-discovered tumors. The goal of surgery should be to remove as much tumor as possible (Level IV-B), whenever feasible and safe, and does not compromise neurological function
Radiotherapy
Immediate RT prolongs the period without progression, but fails to impact overall survival. Subjects with high-risk or symptomatic gliomas (seizures) should be the ones for whom immediate RT should be contemplated. The administered dose should be between 45 and 54 Gy, except for very high-risk tumors, such as IDH wild type, which may require doses of up to 60 Gy (Level I-B)
Systemic treatment
The standard of postoperative care for patients with high-risk LGG is RT followed by PCV polychemotherapy (Level I-A)
Vorasidenib is the treatment of choice in patients who were not treated with chemotherapy or radiotherapy and have a tumor remnant after surgery or have had disease regrowth after surgery (Level I-A). Despite this evidence, Vorasidenib is still awaiting approval from FDA and EMA
